# The Effects of “VelaMente?!” Project on Social Functioning of People With Severe Psychosocial Disabilities 

**DOI:** 10.2174/1745017901713010220

**Published:** 2017-11-24

**Authors:** Federica Sancassiani, Stefano Lorrai, Giulia Cossu, Alessio Cocco, Giuseppina Trincas, Francesca Floris, Gisa Mellino, Sergio Machado, Antonio Egidio Nardi, Elisabetta Pascolo Fabrici, Antonio Preti, Mauro Giovanni Carta

**Affiliations:** 1 Department of Medical Sciences and Public Health, University of Cagliari, Cagliari, Italy.; 2 Center of Liaison Psychiatry and Psychosomatic, University Hospital, , Italy.; 3 Laboratory of Panic and Respiration, Institute of Psychiatry of Federal University of Rio de Janeiro (IPUB/UFRJ), , Brazil; 4 Physical Activity Neuroscience, Physical Activity Sciences Postgraduate Program - Salgado de Oliveira University, , Brazil

**Keywords:** Sailing, Sport, Physical activity, Personal recovery, Psychosocial rehabilitation, Social functioning, Stigma

## Abstract

**Introduction:**

Physical activity helps to improve several clinical outcomes of people with severe psychosocial disabilities. The aims of this study were; 1) to assess the efficacy of a psychosocial rehabilitative intervention focused on sailing in a crew on: a) social functioning; b) severity of the psychosocial disability; c) general functioning; d) dysregulation of biorhythms of people with severe psychosocial disabilities, and 2) to evaluate the attenders’ satisfaction about the project.

**Methods:**

A randomized waitlist controlled trial with parallel groups was carried out involving 51 people with severe psychosocial disabilities. The intervention was a 3 months-lasting course to learn sailing in a crew. Just after the randomization, a group began the sailing course and the other group (wait list) attended the sailing course after 3 months of treatments as usual. Before and after the sailing course, as well as the waiting list period, all attenders were assessed by HoNOS, GAF, CGI-S and BRIAN. At the end of the sailing course, they completed also a self-report satisfaction questionnaire.

**Results:**

Social functioning significantly improved after the sailing course (HoNOS total score “time X group”: p=0.011), mainly because of the improvement of psychopathological symptoms (HoNOS symptoms score “time X group”: p=0.003). Furthermore, participants greatly appreciated the rehabilitative program based on sailing in a crew.

**Conclusions:**

When compared to more traditional rehabilitative activities that are usually carried out in mental health services, a psychosocial rehabilitative intervention based on sailing in a crew significantly improve the social functioning of people with severe psychosocial disabilities.

## 1 INTRODUCTION

People with a severe psychosocial disability have a 20-years shorter lifespan than peers without it, mainly because of a sedentary lifestyle, low physical activity, obesity or poor diet, and the long-term consequences of the side-effects of antipsychotic drugs, such as higher cardiovascular and dysmetabolic risk [1-4]. Furthermore, they often show a difficult adherence to treatments, worsened also by the stigmatization about their disability [5, 6]. Overall, patients with severe psychosocial disability often show a complex mixture of clinical conditions strongly related to their lifestyles and social needs [3, 7].

The needs of people with psychosocial disabilities are often referred to as “a lack of” [8]: health, psychosocial wellbeing, access to appropriate forms of care, specific activities performed by a professional [8, 9]. Social isolation is another common phenomenon closely linked with the experience of suffering from a severe psychosocial disability, accentuated also by hypo-stimulating or alienating environments [10]. However, in the mental health promotion field, the concept of “needs” also concerns “a drive for”: participation, satisfaction, personal growing and development during life, mainly in terms of social inclusion and interpersonal relationships’ enhancement [11-16]. From this perspective, the satisfaction about mental health cares expressed by the users could be considered as a useful indicator, able to reflect, in part, their social needs and personal recovery [15-19].

Recent systematic reviews and meta-analyses pointed out that interventions focused on physical activities and sports help to improve the social functioning and other health outcomes of people with schizophrenia [20-22], depression [23-28] and anxiety [29, 30]. There is evidence that in people with severe psychosocial disabilities aerobic exercises, strength exercises, relaxation training, basic body awareness exercises, exercise counseling, sports, lifestyle interventions or a combination of these, significantly improve mental wellbeing when run in routine community settings [31], psychopathological symptoms, such as depressive or psychotic ones [32], improve quality of life, and reduce weight and dysmetabolic risk related to side-effects of medications [22, 32].

It should be noted that in people with severe psychosocial disabilities the dysmetabolic risk could be also related to a dysregulation of biological and social rhythms [33], which often worsens their clinical conditions and daily life [34-37]. As a matter of fact, there are mutual interactions between biological rhythms and their correspondent physiological functions (*i.e.* eating and sleep), on one side, and physical activities and tasks, on the other side [38].

Hence, care for people with severe psychosocial disabilities should include a focus on improving fitness by exercises and physical activities by qualified healthcare professionals, able to motivate and support them in maintaining an active lifestyle [39, 40].

In order to confirm and extend the findings of a previous study about the efficacy of sailing on psychopathological symptoms and social functioning in people with severe psychosocial disabilities [41, 42], our group established the project “VelaMente?!”, a rehabilitative intervention focused on the promotion of physical activity (sailing in a crew) in people with severe psychosocial disabilities [43]. The main findings of the previous study [41, 42] concerned a statistically significant improvement of the clinical status (p < 0.0001, assessed by Brief Psychiatric Rating Scale - BPRS), the social functioning (p < 0.0001, assessed by HoNOS Scale) and the quality of life (p < 0.001, assessed by WHOQOL-Bref) of people with severe psychosocial disabilities attending a rehabilitative intervention focused on sailing when compared to a control group.

As already pointed out [43], the main differences between the current and the past study [41, 42] are: the enrollment of a larger sample for the intervention (N 64 instead of N 40); the promotion of the attenders’ social skills in a supportive and no-stigmatizing environment, such as a sailing school sited in a popular touristic harbor and the contiguous open sea; and the focus on a more structured group physical activity (a 3 months-lasting course to learn sailing in a crew adapted to the needs of each participant, with the employment of sailing teachers and mental health operators; the choice of two regular sailboats usually used by professional sailors’ crews).

This paper reports the results of the trial according to the following aims of “VelaMente?!” project:

* Primary aim: to assess the efficacy of a psychosocial rehabilitative intervention focused on sailing in a crew on these main outcomes: a) social functioning; b) severity of the psychosocial disability; c) general functioning and d) dysregulation of biorhythms (sleep, eating, social and daily activities) of people with severe psychosocial disabilities;

* Secondary aim: to evaluate the attenders’ satisfaction about the project and the sailing course.

## 2 METHODS

### 2.1 Study Sample and Design

The methodology (randomized waitlist-controlled trial with parallel groups; inclusion/exclusion criteria for recruitment; randomization procedure; assessment timing) and the interventions were already described in detail in the companion publication about the psychosocial rehabilitative program “VelaMente?!” [43].

**Fig. (1):**
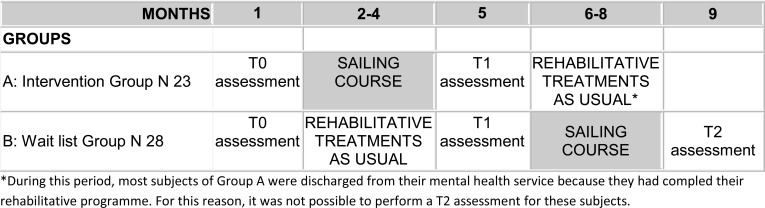
Study design.

A short synthesis is reported below, together to the study design (Fig. **1**).

Out of 64 people with severe psychosocial disabilities (Schizophrenia, Affective Psychosis, and/or Personality Disorder diagnosis according to ICD-X [44]), a total of 51 subjects accepted to take part in the study and were randomly assigned to:

Group A: N 23 subjects enrolled into a 3 months-lasting sailing course plus drugs treatments as usual. Once the sailing course ended, they came back to the usual rehabilitative activities in their mental health service plus drug treatment as usual;

Group B: N 28 subjects enrolled in a wait-list and that attended the usual rehabilitative activities in their mental health service plus drug treatment as usual during the first 3 months. Once the Group A finished the sailing course, they started with the experimental intervention plus drugs treatments as usual.

The experimental treatment (“sailing”) was a psychosocial rehabilitative intervention focused on a structured, 3 months-lasting course to learn sailing in a crew. Each lesson was attended by a group of 8-10 subjects and carried out by two skippers, two psychologists and two trainees attending the “Techniques for the psychiatric rehabilitation” Degree Course of the University of Cagliari, Italy. Lessons took place 2 time/week at a sailing school sited in a touristic harbor in the Gulf of Cagliari. The attenders reached the harbor from their mental health services by a caravan driven by a mental health operator. The sailing course included theoretical sessions and exercises about sailing principle/techniques that were carried out in the open sea or in the harbor by two regular sailboats.

The rehabilitative treatment as usual (“rTAU”) was a mixture of self-help groups, group-supported work in the garden, group-laboratories of art therapy, usually offered in the mental health services.

### 2.2 Assessment Tools

*Ad hoc* questionnaire: To collect the socio-demographic and usual cares information, as well as the clinical status (see Table **1** for details).

*Health of the Nation Outcome Scale* (HoNOS) [45] in the Italian version [46, 47]: to measure social functioning in terms of problems related to suffer from a psychosocial disability. The HoNOS is a clinical scale filled out by the treating clinician. It includes 12 items to evaluate four domains, corresponding to the kind of problems that negatively impact the social functioning: “behavioral”, “cognitive and physical impairment”, “psychopathological symptoms” and “social”. Each item is scored by a 5 points Likert scale, ranging from 0 = “no problem” to 4 = “severe/very severe problems”.

It is synthetized in a total score, ranging from 0 to 48, and a score for each domain (“behavioral”: range 0-12; “cognitive and physical impairment”: range 0-8; “psychopathologica symptoms”: range 0-12; “social”: range 0-16), with higher scores indicating lower levels of social functioning.

The Italian version of the *Global Assessment of Functioning* (GAF) [48]: to measure the general functioning. The GAF is a brief scale completed by the treating clinician. Its scores range from 0 to 100, with higher scores indicating higher levels of functioning.

The Italian version *Clinical Global Impression – Severity scale* (CGI-S) [49]: to assess the severity of the psychosocial disability. The CGI-S is a 7-point Likert scale that requires the rate of the treating clinician at the time of assessment. The treating clinician, on the bases of his/her global experience as clinician, assesses the patient as: 1 = “normal, not at all ill”; 2 =“borderline mentally ill”; 3=“mildly ill”; 4=“moderately ill”; 5=“markedly ill”; 6=“severely ill”; 7=“extremely ill”.

*Biological Rhythms Interview of Assessment in Neuropsychiatry* (BRIAN) [50] in the Italian version [51]: to measure the dysregulation of biorhythms. It is an interviewer-administered instrument including 21 items that evaluate five domains: “sleep”, “eating”, “activity”, “sociality” and “predominant rhythm”. All items were rated using a 4 points Likert scale scored 1 = not at all, 2 = seldom, 3 = sometimes, 4 = often, or in some items 1 = never, 2 = seldom, 3 = often, 4 = always, with higher scores indicating higher levels of dysregulation.

As shown in Fig. (**1**), for the intervention group the assessment points were: pre-treatment (T0) and post-treatment evaluation (T1), before and after the sailing course; for the wait list group, the assessment points were: pre- (T0) and post- (T1) waiting list period (rTAU), and post-treatment (T2) at the end of sailing course.

Finally, an *ad hoc* self-report questionnaire was built by an expert panel and used to provisionally evaluate the participants’ satisfaction level about the sailing course. It was administered to all participants at the end of each sailing course. It includes 8 items with Likert scales to assess several aspects about the project “VelaMente?!” (overall judgment; usefulness; clearness of the contents; organizational support; operators’ support; expectations; effects on general health), 2 items with yes/no answer (will to repeat the sailing course; will to suggest the sailing course to a friend), 1 item with an open answer to eventually indicate some suggestions to improve the sailing course.

### 2.3 Statistical Analysis

Regarding pre- and pos-treatment assesments, the subjects attending the sailing course (sailing group) were compared to the subjects in the wait list (rTAU group).

**Fig. (2):**
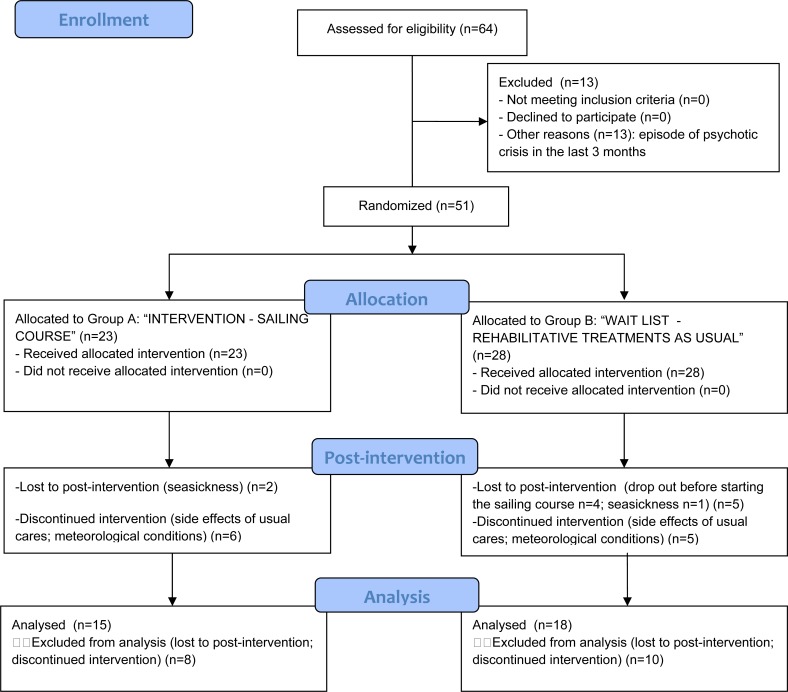
CONSORT Flow Diagram.

Those subjects who were discontinuous during the sailing course (>4 lacked lessons) were excluded from the analyses to have a more precise measure of the rehabilitative effectiveness of the sailing course (Fig. **2**).

The Statistical Package for Social Science (SPSS) for Windows (Chicago, Illinois, 60606, USA), version 21 was used to carry out the data analysis.

The descriptive statistics were performed for nominal (%) and continuous (mean ± SD) variables to describe the socio-demographic and clinical features of the samples (age; gender; marital status; education; diagnosis; psychosocial variables), as well as the level of satisfaction about the sailing course expressed by the participants.

The homogeneity between the two groups at T0 about all variables was tested by a series of Chi-Square (nominal variables) and one-way ANOVA (continuous variables).

The expected differences b y “group”, “time” and “time x group” for the dependent variables (“social functioning based on problems related to the psychosocial disability” (plus domains); “general functioning”; “severity of the psychosocial disability”, “dysregulation of biorhythms”) was tested by a series of repeated measures ANOVA.

### 2.4 Ethical Aspects

The study was approved in November 27, 2014 by the Ethical Committee of the Sardinia Region, Italy and registered with the number “PG/2014/19645”.

A written informed consent was obtained from each attender. All attenders were requested to present a medical certification approving their eligibility for non-agonistic sport activity and they were provided for a health insurance against general accidents related to the sport practice.

## 3 RESULTS

### 3.1 Socio-Demographic, Clinical and Psychosocial Characteristics of The Samples

Table (**1**) shows the socio-demographic, clinical and psychosocial characteristics of both study samples at T0. No statistically significant difference was found about gender, age, marital status, education, diagnosis, social functioning, general functioning, severity of the psychosocial disability, dis-regulation of biorhythms in the comparison of the “sailing” group to the “ rTAU” (wait list) group at T0.

### 3.2 Drop-out and Discontinuity During the Sailing Course Attendance

Overall, N 2 participants in Group A and N 1 in Group B dropped out because seasickness; additional 4 participants in Group B were lost before starting the sailing course (Fig. **2**).

There were N 6 participants in Group A and N 5 participants in Group B that discontinuously attended the sailing course, mainly due to the side effects of drug therapy or to some changes in the lessons timetable because the meteorological conditions.

No statistical difference about outcome measures (CGI-S, HoNOS, GAF, BRIAN) between these subjects and those included in data analyses was detected at T0.

### 3.3 Outcome Measures

HoNOS – social functioning problems related to the psychosocial disability.

As shown in Table (**2**), there was a statistically significant improvement on social functioning in terms of problems related to the psychosocial disability (HoNOS tot; “group”: p = 0.888; “time”: p = 0.001; “time x group”: p = 0.011).

There was also a statistically significant improvement on problems due to the psychopathological symptoms, with a greater decrease in their level for the sailing group than in the wait list group (HoNOS sympt: “group”: p = 0.906; “time”: p = 0.0001; “time x group”: p = 0.003).

Even if there was no statistically significant “time x group” interaction on social problems, there was a more evident decrease in their level for the sailing group than in the wait list group (HoNOS soc: “group”: p = 0.930; “time”: p = 0.039; “time x group”: p = 0.189).

Finally, there was no statistically significant difference about problems due to the physical and cognitive impairment (even if a very low improving trend in the sailing group) and behavioral problems, that tend to slowly improve in both groups.

GAF – general functioning

There was a statistically significant improvement on general functioning over time, (GAF: “group”: p = 0.677 “time”: p = 0.001; “time x group”: p = 0.059).

CGI-S – severity of the psychosocial disability

Even if there was no statistically significant “time x group” interaction in the severity of the psychosocial disability, there was a trend for improvement in the wait list group and a relative stability in the sailing group (CGI-S: “group”: p = 0.749; “time”: p = 0.010; “time x group”: p = 0.553).

BRIAN –dysregulation of biological and social rhythms

There was no statistically significant difference by group or over time in the measure of the dysregulation of biological and social rhythms (BRIAN: p > 0.05).

### 3.4 Satisfaction Levels After the Sailing Course

As shown in Table (**3**), among the sailing course attenders that filled in the questionnaire (n = 33), 17 (51.0%) judged “very good” the “VelaMente?!” project, 9 (27.3%) “good”, 7 (21.2%) “sufficient”, none reported a “bad” or “very bad” judgment.

Twenty participants (60.6%) considered the project as “very usefulness”, 5 (15.2%) “usefulness”, 8 (24.2%) “sufficiently usefulness”, none evaluated it as “useless” or “very useless”.

Just 1 participant (3.0%) “already knew” the contents proposed during the lessons, 25 (75.7%) “did not already know” them, and 7 (21.3%) “knew them just in part”.

There were 22 (66.7%) attenders that considered “very good” the didactic of sailing teachers during the lessons, 9 (27.2%) “good”, 2 (3.1%) “sufficient”, none evaluated it as “bad” or “very bad”. The expectations of the 33 participants about the sailing course were satisfied with a mean level of 71.2 (SD= 21.71; out of a range of rating from 0 to 100).

Further details in the Table **3**.

There were 31 (93.9%) participants that considered “good” the availability for support and listening from all the operators involved during the lessons, 2 (6.1%) “sufficient”, none considered it “bad”.

There were 19 (57.6%) participants that considered that the sailing course improved their general health, 3 (15.1%) attenders did not spot this effect, 9 (27.3%) spotted it, but just in part.

The expectations of the 33 participants about the sailing course were satisfied with a mean level of 71.2 (SD= 21.71; out of a range of rating from 0 to 100); 30 (90.9%) attenders would like to repeat the experience, 3 (9.1%) of them would not. Finally, all participants would like to suggest the experience to a friend.

## 4 DISCUSSION

The main purpose of this study was to evaluate the efficacy of a psychosocial rehabilitative intervention focused on a course to learn sailing in a crew on some clinical outcomes of people with severe psychosocial disabilities. It was considered the social and general functioning, the severity of the psychosocial disability and the dysregulation of biorhythms. The general hypothesis was that these outcomes are liable to improvement by attending a structured physical activity program [13, 14, 16, 21-25, 27-32, 39-42, 56-58].

Indeed, findings pointed out that the social functioning significantly improved after the sailing course when compared to more traditional psychosocial rehabilitative group activities (self-help, gardening, art therapy). This improvement regarded almost all problems related to suffering from a psychosocial disability with negative impact on the social functioning, in particular those due to psychopathological symptoms.

Regarding the general functioning, there was an improvement in both groups but without any significant difference between the wait list and the sailing group.

The dysregulation of the biorhythms remained almost stable over time in both groups, when measured on the BRIAN range scores. Essentially, the biorhythms did not benefit from the improvement in social and psychopathological problems brought about by the “VelaMente?!” program. It could be speculated that biorhythms (eating, sleep, daily and social activities) in both groups were already well stabilized by the treatment as usual.

We found a high level of appreciation and satisfaction reported by attenders at the end of the sailing course. Even if it was not possible to compare these results with those of a control group, it is known that good levels of patient-reported mental, physical and general health predict their higher satisfaction about cares [17]. This is a crucial point in the mental health promotion field, mainly when it was proposed an innovative rehabilitative intervention such as “VelaMente?!” oriented to the promotion of personal recovery of people with severe psychosocial disabilities [19], to fight against cultural stigma around their illness [5] and specifically aimed to improve participants’ social and general functioning, the severity of the psychosocial disabilities and a high level of their satisfaction about the intervention.

The efficacy of social contacts-based interventions in improving people with psychosocial disabilities’ wellbeing [52], such as community-based exercise projects [31], is well known. However, a systematic review and meta-analysis [55], including 8 RCTs out of 216 studies assessed for eligibility, pointed out that exercise therapies for people with severe psychosocial disabilities can lead to a modest increase in levels of exercise activity but without any changes for psychopathological symptoms. Hence, authors concluded that change brought about by exercise might be more easily achievable within a more closely supervised setting such as an inpatient ward [55].

The findings of the present study are coherent with those pointed out by another systematic review [56] which stated that, when compared with indoors physical activities, exercising in natural environments was associated with greater feelings of revitalization and positive engagement, lower tension, confusion, anger, and depression, with higher energy, greater enjoyment, satisfaction and intent to repeat again the activity.

The course to learn sailing in a crew proposed by “VelaMente?!” project had two peculiar features: a) it was conducted by sailing teachers and facilitated by mental health operators, and, b), lessons took place at the harbor and the sea, relatively far from the mental health services where usually recruited subjects were treated for their usual cares [43]. Hence, as already pointed out [41, 42, 56], it is possible that this kind of outdoor physical activity intervention could positively impact the daily management of the psychosocial disability, in particular as far as psychopathological symptoms, improving social functioning and enhancing great levels of satisfaction about cares in people with psychosocial disabilities were concerned.

## 5 LIMITATIONS

The present study shows some limits. Simple multivariate analyses was performed because the small sample size. For the same reason, it was decided to avoid to assess and to control for variables such as subjects' cognitive level and how long they were in charge into the mental health services. However, the parallel groups design with one group crossed to the experimental intervention, partially moderated the limits due to the small sample size because many subjects serve as their own controls, restraining the effects of confounding factors.

The discharge of a high number of subjects involved in the first group (Group A) among those who attended the sailing course, due to the end of the rehabilitation program in their mental health services, did not allow assessing them three months after the post-treatment assessment (T2). This substantial attrition rate did not allow obtaining the follow-up measures necessary to provide information about longevity of outcomes and lasting effects of interventions, as well as to perform a cross-over design.

Finally, the use of control interventions that are not evidence based may have determined some bias in favor of the experimental intervention.

## CONCLUSION

The findings of the present study highlight that, when compared to more traditional group psychosocial rehabilitative activities, a structured outdoor physical activity program focused on sailing in a crew significantly improves social functioning of people with severe psychosocial disabilities. Furthermore, the high satisfaction expressed by the attenders about this kind of psychosocial rehabilitative program could reflect the realization of a personal recovery-centered intervention with positive impacts on cultural stigma about psychosocial disabilities.

Findings of the present study, even if with their limits, contribute to show that there is a great potential to develop innovative, promising and effective programs in the psychosocial rehabilitation field.

## Figures and Tables

**Table 1:** Socio-demographic and clinical characteristics of the samples at T0.

**VARIABLES**	**DESCRIPTIVE STATISTICS**		**HOMOGENEITY STATISTICS**
	**SAILING ** **GROUP** **(N 33)**	**WAITING LIST (rTAU) GROUP** **(N 18)**	
Socio-demographic			
**Gender** M F	27 (81.8%) 6 (12.2%)	13 (72.2%) 5 (27.8%)	Chi-Square = 0.194, df =1, p = 0.6599
**Age**	37.76±11.6	36.22±9.17	F = 0.236, df = 1;49, p = 0.629
**Education **(years)	9.3±2.7	9.2±3	F = 0.015, df = 1;49, p = 0.904
**Marital status**	5 (15.2%) 28 (84.8%)	1 (5.6%) 17 (94.4%)	Chi-Square = 0.316, df =1, p=0.5743
(married) yes no			
Clinical			
**Diagnosis**	3 (9.1%) 20 (60.6%) 10 (30.3%)	3 (16.7%) 10 (55.6%) 5 (27.7%)	Chi-Square = 0.6439, df =1, p=0.7247
Schizophrenia spectrum psychoses Affective psychoses Personality disorders			
**Problems related to** ** psychosocial disability**	8.09±5.838 1.27±1.701 0.91±1.100 2.58±1.714 3.33±3.189	7.22±5.331 1.22±1.896 0.94±0.873 2.17±1,855 2.89±2,166	F = 0.274, df = 1;49, p = 0.603 F = 0.009, df = 1;49, p = 0.923 F = 0.014, df = 1;49, p = 0.907 F = 0.626, df = 1;49, p = 0.433 F = 0.278, df = 1;49, p = 0.600
HoNOS (tot) HoNOS (beh) HoNOS (imp) HoNOS (symt) HoNOS (soc)			
**Global ** **Functioning**	63.61±10.170	62.89±9.791	F = 0.059, df = 1;49, p = 0.808
GAF			
**Severity of the** **psychosocial disability**	3.18±0.882	3.17±0.857	F = 0.004, df = 1;49, p = 0.953
CGI-S			
**Biorhythms ** **Dis-regulation**	40.12±6.122	41±8.664	F = 0.178, df = 1;49, p = 0.675
BRIAN (tot)			

Legend

M=male

F= female

tot= total

beh= behavior

imp= impairment

symp= symptoms

soc= social

rTAU: rehabilitative treatment as usual.

**Table 2:** Changes over time in measures of problems related to the psychosocial disability, global functioning, severity of the psychosocial disability, dysregulation of biorhythms.

		**Sailing Group** **N 33**		**Waiting list (rTAU) Group** **N 18**		**Repeated measures ANOVA**		
**Outcomes**	**Measure**	**Pre-** **Treatment** **(mean ± sd)**	**Post-treatment** **(mean ± sd)**	**Pre-treatment** **(mean ± sd)**	**Post-treatment** **(mean ± sd)**	**Group**	**Time**	**Time** **x** **group**
**Social functioning**	HoNOS tot	8.09 ± 5.838	5.45 ± 4.950	7.22 ± 5.331	6.78 ± 6.744	F=0.20 df=1,49; p=0.888	F=13.676 df=1,49 p=0.001	F=6.923; df=1,49 p=0.011
	HonNOS beh	1.27 ± 1.701	1 ± 1.750	1.22 ± 1.896	1.06 ± 1.349	F=0.000 df=1,49 p=0.996	F=1.394 df=1,49 p=0.243	F=0.081 df=1,49 p=0.777
	HoNOS imp	0.91 ± 1.1	0.61± 1.116	0.94 ± 0.873	1.06 ± 1.349	F=0.644 df=1,49 p=0.426	F=0.572 df=1,49 p=0.453	F=2.664 df=1,49 p=0.109
	HoNOS symp	2.58 ± 1.714	1.42 ± 1.458	2.17 ± 1.855	1.94 ± 1.798	F=0.014 df=1,49 p=0.906	F=21.822 df=1,49 p=0.0001	F=2.664 df=1,49 p=0.109
	HoNOS soc	3.33 ± 3.189	2.36 ± 2.460	2.89 ± 2.166	2.67± 3.597	F=0.008 df=1,49 p=0.930	F=4.502 df=1,49 p=0.039	F=1.771 df=1,49 p=0.189
**General functioning**	GAF	63.61±10.170	65.24±9.931	62.89±9.791	68.28±10.087	F=0.175 df=1,49 p=0.677	F=13.101 df=1,49 p=0.001	F=3.738 df=1,49 p=0.059
**Severity of psychosocial disability**	CGI-S	3.18 ± 0.882	2.97 ± 0.81	3.17 ± 0.857	2.83 ± 0.985	F=0.104 df=1,49 p=0.749	F=7.221 df=1,49 p=0.010	F=0.357 df=1,49 p=0.553
**Dysregulation of biorhythms**	BRIAN	40.12 ± 6.122	40.58±7.822	41 ± 8.664	40 ± 7.799	F=0.006 df=1,49 p=0.938	F=0.068 df=1,49 p=0.795	F=0.485 df=1,49 p=0.490

Legend

sd = standard deviation

tot = total

beh = behavior

imp = impairment

symp = symptoms

soc = social

rTAU: rehabilitative treatment as usual

**Table 3:** Participants’ satisfaction about the “VelaMente?!” project.

Questionnaire item	**Answers** **N (%)**
Overall, how do you judge the “VelaMente?!” project?	Very good: 17 (51.5) Good: 9 (27.3) Sufficient: 7 (21.2) Bad: 0 (0) Very bad: 0 (0)
How about the usefulness of the “VelaMente?!” project?	Very useful: 20 (60.6) Useful: 5 (15.2) Sufficiently: 8 (24.2) Useless: 0 (0.0) Very useless: 0 (0.0)
How about the contents proposed during the sailing course?	I already knew them: 1 (3.0) I knew them just in part: 7 (21.3) I did not already know them: 25 (75.7)
How about the clearness of the sailing teachers during the lessons?	Very good: 22(66.7) Good: 9 (27.2) Sufficient: 2 (3.1) Bad: 0 (0.0) Very bad: 0 (0.0)
How about the organizational support during the sailing course? (*i.e.*: dedicate room, boats, tools, *etc*. …)	Very good: 19 (57.6) Good: 12 (36.4) Sufficient: 2 (6.0) Bad: 0 (0.0) Very bad: 0 (0.0)
How about the availability for support and listening of the operators (sailing teachers and mental health operators) during the sailing course?	Good: 31 (93.9) Sufficient: 2 (6.1) Bad: 0 (0.0)
In your opinion, the sailing course improved your general health?	Yes: 19 (57.6) In part: 9 (27.3) No: 3 (15.1)
In which percentage your expectations about the sailing course were satisfied?	From 0% to 100% (N 33, mean ± sd): 71.2 ± 21.71
Would you like to give us any suggestions to improve the contents of the sailing course?	Yes: 10 (30.3) No: 23 (69.7)
Would you like to suggest this experience to a friend?	Yes: 33 (100) No: 0 (0.0)
Would you like to repeat again this experience?	Yes: 30 (90.9) No: 3 (9.1)
